# Extraction, purification, structural characterization, and health benefit of the litchi (*Litchi chinensis* Sonn.) polysaccharides: a review

**DOI:** 10.3389/fphar.2025.1618885

**Published:** 2025-08-18

**Authors:** Bo Wang, Haizheng Bi, Mengru Zhang, Xinrui Xu, Meng Wang

**Affiliations:** Heilongjiang University of Chinese Medicine, Key Laboratory of Basic and Application Research of Beiyao, Ministry of Education, Harbin, China

**Keywords:** *Litchi chinensis* Sonn., polysaccharides, extraction, purification, structural characteristics, health benefit

## Abstract

Litchi, a prominent species within the *Litchi Sonn* genus of the Sapindaceae family, is a fruit renowned for its significant nutritional, health, and pharmacological properties. Polysaccharides are one of the functional active ingredients in litchi, with various biological functions such as hypoglycemic effect, prebiotic effect, promoting effect on exopolysaccharide production by *Weissella confuse*, antioxidant effect, antiproliferative effect, immunoregulatory effect. Consequently, research on litchi polysaccharides has steadily expanded and deepened in recent years. A comprehensive review of existing studies is essential for advancing the understanding of these polysaccharides. This article provides an overview of the current state of research on litchi polysaccharides, covering various aspects such as extraction, purification, chemical structure, health benefits, and structure-activity relationships. Furthermore, it offers valuable insights to guide the continued development of litchi polysaccharides, establishing a scientific foundation for their more efficient and rational utilization.

## 1 Introduction

**FIGURE 1:**
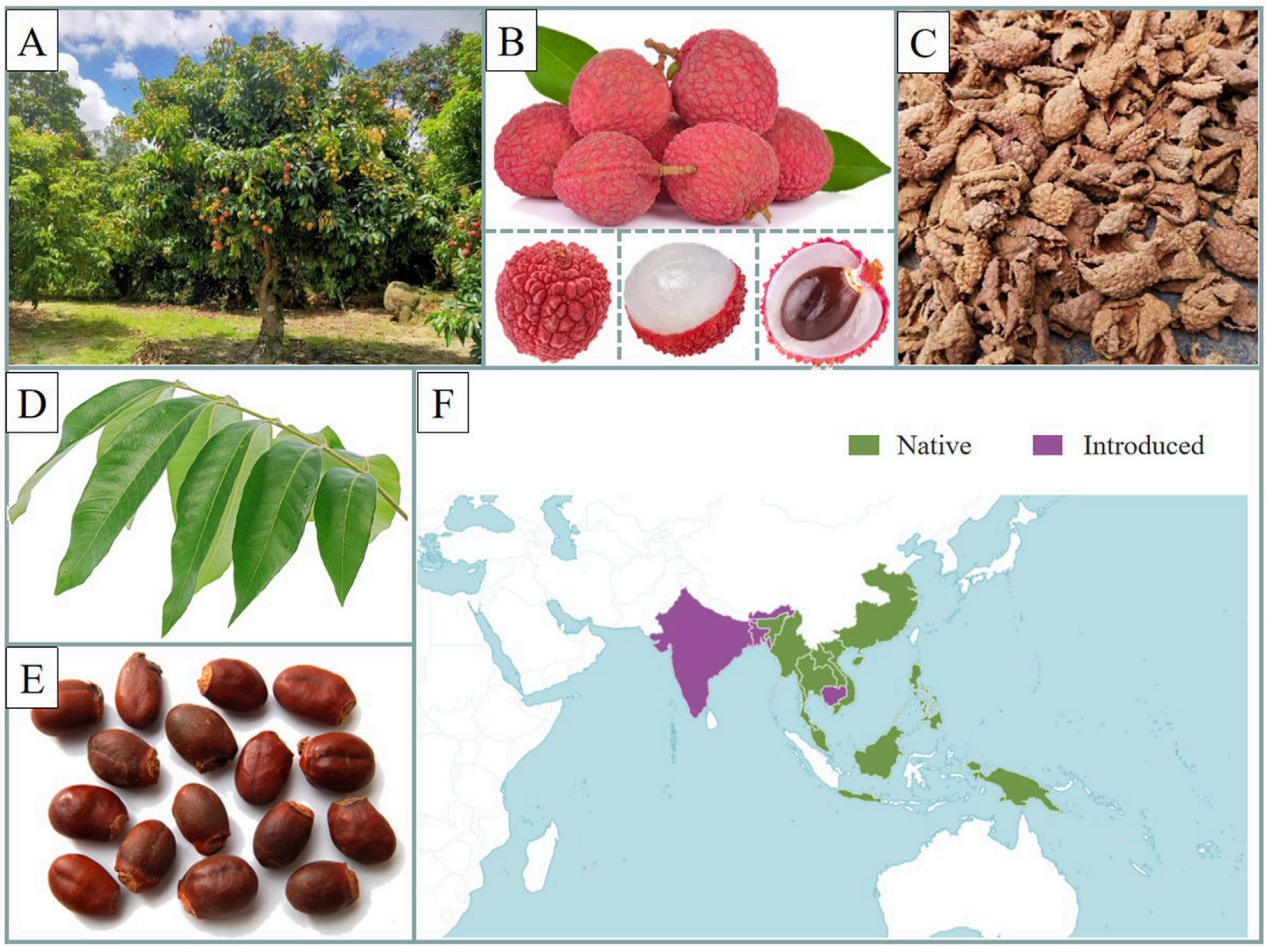
The botanical characteristics of litchi. **(A)** The tree of litchi. **(B)** The fruit of litchi. **(C)** The pericarp tissues of litchi. **(D)** The leaves of litchi. **(E)** The seeds of litchi. **(F)** The distribution of litchi in world. (https://powo.science.kew.org/taxon/urn:lsid:ipni.org:names:30014909-2. 20 April 2025). (Pictures are from public sources and the Internet).

*Litchi chinensis* Sonn., commonly known as litchi, is a fruit-bearing tree in the Sapindaceae family (Bishayee et al., 2024). Native to China, it is now cultivated in regions including Vietnam, Thailand, India, Madagascar, and Mauritius (Yang et al., 2024). Litchi trees can grow up to 15–20 m in height. Its wood is strong, durable, and resistant to corrosion, making it suitable for crafting load-bearing furniture. When this fruit is ripe, its skin is typically red or purple, with scale-like protrusions on the surface. Litchi seeds are oblong or oval, slightly flattened, with a brownish red or purple-brown surface (Emanuele et al., 2017; Chukwuma et al., 2021) (Figure 1). The fruit itself is oval or nearly spherical, sweet, and juicy, offering a refreshing taste. Fresh litchi fruits are consumed directly, and due to their vibrant color and appealing flavor, litchis have gained significant popularity (Cao et al., 2024; Wang D. et al., 2023). Due to their short shelf life, litchis are often referred to as “seasonal fruits,” but to meet demand, they are processed into preserved fruit, jelly, jam, and other products, which retain the original flavor and provide consumers with greater variety.

Litchi is rich in several nutrients, including sugars, organic acids, amino acids, vitamins, dietary fiber, carotenoids, and minerals (Richter Reis et al., 2017; Liu H. et al., 2023). Consuming litchi in moderation offers various health benefits. Its sugars, primarily glucose and fructose, are easily absorbed by the body. The dietary fiber promotes intestinal peristalsis and enhances digestive function, while the vitamin C content bolsters immune health. Additionally, litchi has considerable medicinal value. According to the Chinese Pharmacopoeia (2020 edition), litchi seeds (litchi semen) are used to treat hernia pain and testicular swelling (Liang et al., 2024). In India, litchi seeds are ground into powder for treating intestinal disorders and used in tea to alleviate nerve pain. The flesh of the fruit is beneficial for conditions such as asthma, insomnia, anemia, and palpitations, while the pericarp is used to treat diseases like dysentery and eczema (Zhao et al., 2020; Oulkar et al., 2022). As a “super fruit” with numerous health benefits, litchi is increasingly recognized for its chemical composition and therapeutic potential.

Recent studies have identified a variety of compounds, including polysaccharides, phenols, and flavonoids, isolated from litchi (Huang et al., 2021). Phytochemical and pharmacological research indicates that polysaccharides are among the primary bioactive constituents in litchi (Chen et al., 2023). The health benefits of litchi are related to polysaccharides. Litchi polysaccharides obtained through various extraction and purification methods have novel structural features and multiple physicochemical properties. These polysaccharides demonstrate significant biological activities and health benefits, such as hypoglycemic effect, prebiotic effect, promoting effect on exopolysaccharide production by *Weissella confuse*, antioxidant effect, antiproliferative effect, immunoregulatory effect (An et al., 2022; He et al., 2022; Hu et al., 2023; Zou et al., 2023). Moreover, the non-toxic and biocompatible nature of litchi polysaccharides has positioned them as a focal point in basic research and practical applications in functional foods, cosmetics, health products, and pharmaceuticals.

Although numerous reviews on litchi exist, most focus on small-molecule compounds. To date, no systematic and comprehensive review has been published specifically on litchi polysaccharides, a gap that limits their further development, utilization, and understanding by both basic researchers and industries in the food and pharmaceutical sectors. This article offers an in-depth and systematic review of the latest advancements in the extraction, purification, structural characterization, and health benefits of litchi polysaccharides, while also exploring the relationship between their structural features and biological activities. The goal is to encourage further research into litchi polysaccharides and provide valuable insights and resources for researchers in this field.

## 2 Extraction and purification of litchi polysaccharides

**FIGURE 2:**
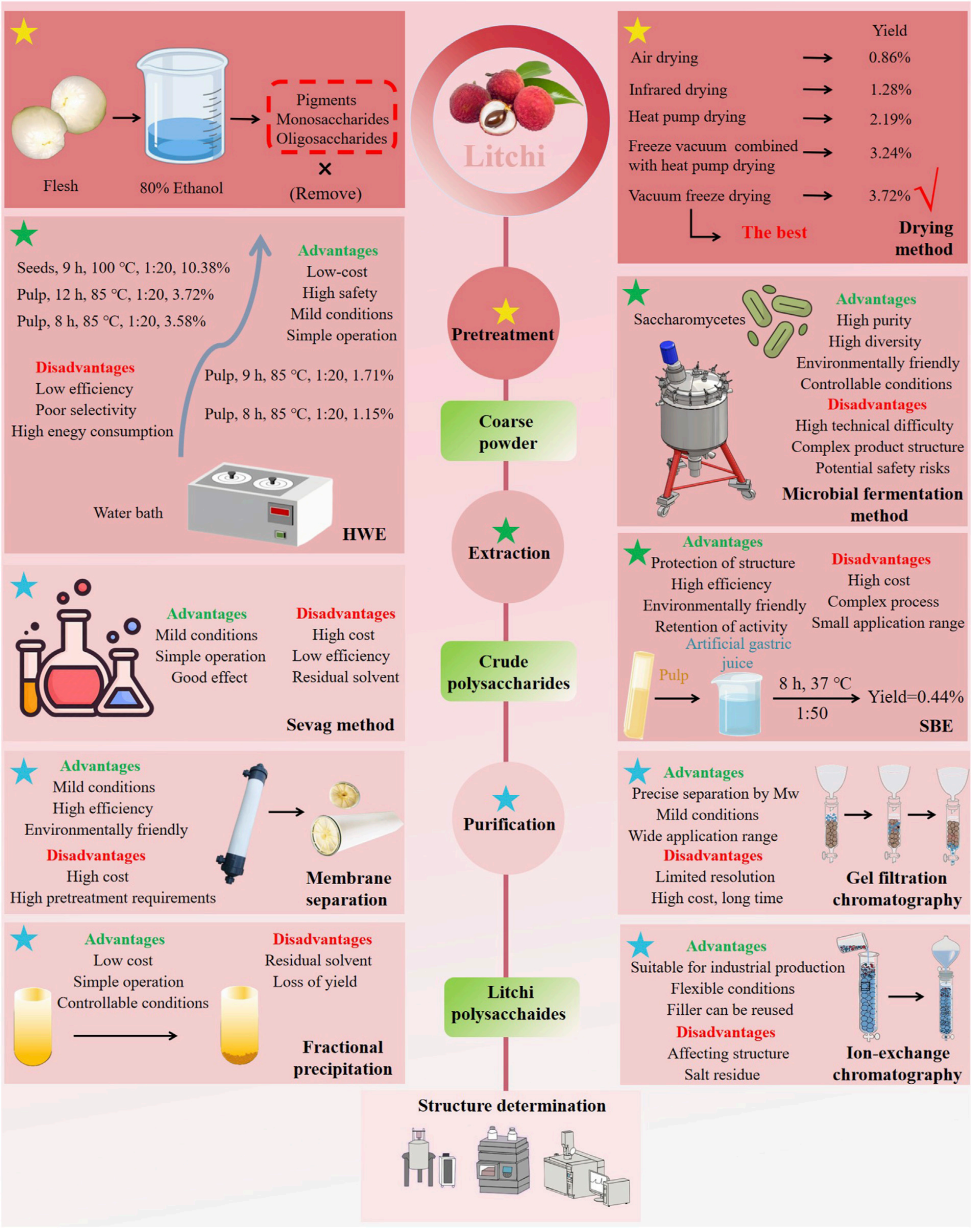
Regarding the extraction and purification of litchi polysaccharides (Pictures are from public sources and the Internet).

Plant polysaccharides predominantly reside in the cell wall and cytoplasm of plants (Guo et al., 2024). The selection of an appropriate pretreatment method is essential before extraction, based on the form and source of the polysaccharides (Khamassi and Dumon, 2023; Zhang et al., 2023) (Figure 2). For instance, plant seeds, which are rich in lipids, must undergo treatment with lipophilic solvents to remove fats prior to polysaccharide extraction. Similarly, when extracting non-starch polysaccharides from plants like grains and legumes, amylase treatment is necessary to eliminate starch before proceeding with polysaccharide extraction. In the case of litchi polysaccharides, the primary extraction site is the flesh. Typically, 80% ethanol is used prior to extraction to eliminate pigments, monosaccharides, and oligosaccharides. Drying is also a critical pretreatment step, as litchi is highly perishable. The choice of drying method is crucial for preservation, and it plays a key role in maintaining the physicochemical and pharmacological quality of the extracted polysaccharides (Cao et al., 2020). An et al. (2022) investigated the effects of five different drying methods on LPPs (litchi polysaccharides). The polysaccharides obtained from these methods, designated LPPA, LPPI, LPPH, LPPF, and LPPFH, exhibited yields of 0.86%, 1.28%, 2.19%, 3.72%, and 3.24%, respectively, in descending order of LPPF > LPPFH > LPPH > LPPI > LPPA. Vacuum freeze-drying resulted in the highest yield, while air drying produced the lowest. Additionally, drying methods were found to influence the biological activity of the polysaccharides. This highlights the importance of pretreatment in polysaccharide extraction, with improvements in these processes potentially increasing extraction yield and purity, thus lowering production costs.

After the pretreatment is completed, the next step is to start extracting polysaccharides. There are many methods for extracting polysaccharides currently available. Hot water extraction (HWE), known for its low cost and ease of operation, is commonly employed for extracting litchi polysaccharides (Cheng et al., 2024). The extraction typically lasts 2–6 h, with temperatures ranging from 30°C to 95°C, although 85°C is most commonly used. The yield of polysaccharides using this method ranges from 1.15% to 10.38% (Table 1). HWE takes advantage of the fact that polysaccharides are insoluble in ethanol but dissolve in hot water. An alternative approach is microbial fermentation, which utilizes microorganisms to metabolize the carbon sources, nitrogen sources, and inorganic salts in the raw material during growth. This method does not rely on the solubility characteristics of polysaccharides but instead achieves their purification and enrichment (Li et al., 2014). Microbial fermentation offers the advantage of shorter extraction times, lower production costs, and enhanced polysaccharide yield efficiency. Hu et al. (2023) combined 24 kg of litchi juice with 30 mg/mL of active yeast in a sterile fermentation tank, fermenting at 25°C to deplete glucose, sugar, and fructose from the juice. The supernatant was then collected, centrifuged, and polysaccharides were extracted *via* alcohol precipitation. Additionally, the semi-bionic extraction (SBE) method, which mimics the gastrointestinal transport and absorption environment of oral drugs, has been employed for polysaccharide extraction. Wang et al. (2015) utilized the SBE method to extract litchi polysaccharides (LCPA50), wherein the polysaccharides were soaked in artificial gastric juice, extracted at 37°C for 4 h, repeated twice, yielding a polysaccharide content of 0.44%. Extraction methods and technologies for plant polysaccharides are continuously evolving, though each method should be adapted and investigated based on the specific properties and characteristics of the target polysaccharides. Comprehensive comparisons should be made when selecting extraction and separation techniques, and experiments must be conducted to identify the most effective method and process.

**TABLE 1:** A summary of litchi polysaccharides extraction and purification methods.

Part of the plant	Extraction						Purification		References
	Polysaccharide fraction	Extraction methods	Time (h)	Temperature (°C)	Solid–liquid ratio	Polysaccharide yield (%)	Polysaccharidefraction	Purification methods	
The pericarp tissues of litchi	F1	HWE	2 h × 3	30°C	1:20	NA	F01	DEAE Sepharose fast-flow column, Sephadex G-50 gel column	Yang et al. (2006)
Litchi juice	CLzp1, CLzp2	Microbial fermentation method	NA	25°C	NA	NA	Lzp1 and Lzp2	DEAE-cellulose 52	Hu et al. (2023)
	Lzp2	Microbial fermentation method	NA	25°C	NA	NA	Lzp2-1 and Lzp2-2	Sephadex G-100	Hu et al. (2023)
Litchi fruits	CLP	HWE	4 h × 2	95°C	1:20	NA	LP1 and LP2	Ultrafiltration membrane (100 kDa)	Zou et al. (2023)
Litchi pulp	LPPA	HWE	6 h × 2	85°C	1:20	0.86%	NA	NA	An et al. (2022)
	LPPI	HWE	6 h × 2	85°C	1:20	1.28%	NA	NA	An et al. (2022)
	LPPH	HWE	6 h × 2	85°C	1:20	2.19%	NA	NA	An et al. (2022)
	LPPF	HWE	6 h × 2	85°C	1:20	3.72%	NA	NA	An et al. (2022)
	LPPFH	HWE	6 h × 2	85°C	1:20	3.24%	NA	NA	An et al. (2022)
	LP (H-2018)	HWE	4 h × 2	85°C	1:20	NA	LP-II	DEAE-Sepharose Fast Flow column	Huang et al. (2018), Huang et al. (2019)
	LP (H-2016-a)	HWE	4 h	85°C	N/A	NA	NA	NA	Huang et al. (2016a)
	LP (H-2016-b)	HWE	4 h × 2	85°C	1:20	3.58%	LP1, LP2 and LP3	DEAE 52-cellulose column, Sephadex G-100	Huang et al. (2016b)
	Crude polysaccharide	HWE	NA	85°C	N/A	1.18%	LCP70W and LCP70WS-1	DEAE-cellulose 52, Sephadex G-100	Hu et al. (2015)
	LPFs	HWE	4 h × 2	85°C	1:20	NA	LP-4, LP-6 and LP-8	Graded precipitation at final ethanol concentrations of 40%, 60% and 80%	Huang et al. (2015)
	LPD	HWE	4 h × 2	85°C	1:20	3.58%	NA	NA	Huang et al. (2014)
	LPF	HWE	4 h × 2	85°C	1:20	1.15%	NA	NA	Huang et al. (2014)
	LCP50S	HWE	3 h × 3	80°C	NA	NA	LCP50S-1, LCP50S-2	DEAE-cellulose 52, DEAE-Sepharose Fast Flow columns	Hu et al. (2011)
	LCP50	HWE	3 h × 3	85°C	1:20	1.71%	LCP50W	DEAE-cellulose 52, Sephadex G-100, and Sephacryl S-300 HR column chromatography	Jing et al. (2014)
	LFP-I, LFP-II, LFP-III and LFP-IV	HWE	4 h × 2	80°C	NA	NA	NA	EtOH stepwise precipitation	Kong et al. (2010)
	LCPA50	SBE	4 h × 2	37°C	1:50	0.44%	LCPA50-S1, LCPA50-S2	Ultrafiltration cell with membrane pores of 3,000 Da	Wang et al. (2015)
	NA	HWE	4 h	80°C	1:8	NA	LPPBa	DEAE-Sepharose Fast Flow, Sephacryl S-400HR	Yang et al. (2016)
The seeds of litchi	LSP	HWE	3 h × 3	100°C	1:10	10.38%	LSP-W-4	DEAE-Sepharose Fast Flow columns, Superdex™ G-75 column	Wu et al. (2020)

Abbreviations: HWE, hot water extraction; SBE, semi-bionic extraction; NA, information was not available.

The polysaccharide extract obtained through these methods often contains impurities such as inorganic salts, proteins, lignin, and pigments, which affect purity. Inorganic salts and low-molecular-weight non-polar substances can be removed by dialysis (Shi, 2016). Ion exchange resins are commonly used to eliminate inorganic salts (Paulsen et al., 2002). For protein removal, various methods are available, including enzymatic methods, the Sevag method, and trichloroacetic acid treatment, with the Sevag method being the most widely used for litchi polysaccharides (Yang and Huang, 2023). To separate and purify litchi polysaccharides, techniques such as fractional precipitation, membrane separation, and column chromatography are commonly applied. For example, after protein removal, litchi polysaccharide extracts undergo fractional precipitation with ethanol concentrations of 40%, 60%, and 80%, followed by freeze-drying to obtain LP-4, LP-6, and LP-8 (Huang et al., 2015). Zou et al. (2023) dissolved 100 mg of litchi polysaccharide (CLP) in 20 mL of distilled water and purified it using an ultrafiltration membrane with a 100 kDa molecular weight cutoff, yielding two distinct polysaccharides, LP1 and LP2. Chromatography methods, including ion exchange and gel filtration chromatography, are routinely used for polysaccharide separation and purification (Guan et al., 2024; Li et al., 2024). Common ion exchangers for litchi polysaccharides include DEAE Sepharose FF and DEAE cellulose, typically eluted with distilled water or NaCl solution. For gel filtration, Sephacryl S and Sephadex G series are frequently used and are generally washed with deionized water.

## 3 Chemical compositions and structural characteristics of litchi polysaccharides

The monosaccharide composition, molecular weight (Mw), structure, and spatial configuration of polysaccharides are critical factors influencing their biological activity. For litchi polysaccharides, these characteristics—such as content, structure, Mw, and composition—are influenced by factors like growth conditions, cultivation techniques, and the methods of extraction and purification. The chemical composition and structural features of litchi polysaccharides are summarized in Table 2.

**TABLE 2:** Source, compound name, molecular weights, monosaccharide composition, structures of litchi polysaccharides, and analytical techniques.

Source	Compound name	Molecular weights	Monosaccharide composition	Structures	Analytical techniques	References
The pericarp tissues of litchi	F01	14 kDa	(%) Man: Gal: Ara = 65.6: 33.0: 1.4	(1→2)-glycosidic linkages: 8.7%. (1→3)-glycosidic linkages: 83.3%. (1→6)-glycosidic linkages: 8.0%.	GPC, GC, IR	Yang et al. (2016)
Litchi juice	Lzp2-2	5.54 kDa	Gal: Ara: Glc = 1.61: 1.00: 1.14	Major backbone structure: →3)-*β*-d-Gal*p*-(1→6)-*β*-d-Gal*p*-(1 → 6)-*β*-d-Gal*p*-(1 → 3)-*β*-d-Glc*p*-(1 → 6)- *α*-d-Glc*p*-(1 → 3)-*α*-d-Glc*p*-(1→. Side chain: *α* -l-Ara*f*-(1 → 5)-α-l-Ara*f*-(1 → at the O-3 of →6)-*β*-d-Gal*p*-(1→, *β*-d-Glc*p*-(1 → or *α*-l-Ara*f*-(1 → at the O-6 of →3)-*β*-d-Gal*p*-(1→.	GC-IMS, GPC, HPLC, NMR analysis	Hu et al. (2023)
Litchi fruits	LP1	378.67 kDa	(%) Rha: Ara: Man: Glc: Gal = 0.90 ± 0.04: 25.14 ± 0.17: 29.76 ± 0.12: 13.55 ± 0.13: 30.65 ± 0.21	NA	GC-MS, FT-IR	Zou et al. (2023)
	LP2	16.96 kDa	(%) Rha: Ara: Man: Glc: Gal = 5.03 ± 0.12: 50.73 ± 0.31: 7.58 ± 0.08: 4.49 ± 0.01: 32.17 ± 0.23	NA	GC-MS, FT-IR	Zou et al. (2023)
Litchi pulp	LPPA	Fraction I: 85.60 kDa. Fraction II: 5.40 kDa.	(%) Rib: Rha: Ara: Xyl: Man: Glc: Gal = 1.99: 5.09: 15.22: 0.51: 11.35: 25.63: 40.21	NA	NA	An et al. (2022)
	LPPI	Fraction I: 85.10 kDa. Fraction II: 5.36 kDa.	(%) Rib: Rha: Ara: Xyl: Man: Glc: Gal = 2.23: 7.76: 13.46: 0.63: 9.12: 22.32: 44.48	NA	NA	An et al. (2022)
	LPPH	Fraction I: 82.10 kDa. Fraction II: 4.83 kDa.	(%) Rib: Rha: Ara: Xyl: Man: Glc: Gal = 3.70: 6.10: 14.88: 2.19: 10.72: 26.37: 36.04	NA	NA	An et al. (2022)
	LPPF	Fraction I: 72.9 kDa. Fraction II: 4.06 kDa.	(%) Rib: Rha: Ara: Xyl: Man: Glc: Gal = 3.31: 9.92: 24.21: 1.25: 9.95: 20.60: 30.76	NA	NA	An et al. (2022)
	LPPFH	Fraction I: 80.7 kDa. Fraction II: 4.91 kDa.	(%) Rib: Rha: Ara: Xyl: Man: Glc: Gal = 3.91: 5.39: 14.41: 4.11: 11.70: 34.28: 26.20	NA	NA	An et al. (2022)
	LP-II	161.24 kDa	(%) Ara: Gal: Rha: Glc = 45.2: 34.7: 16.7: 3.4	LPII mainly consisted of *α*-l-Ara*f*-(1→, →5)-*α*-Ara*f*-(1→ and →3,6)-*β*-d-Gal*p*-(1→.	GC-MS, FT-IR, NMR	Huang et al. (2019)
	LP (H-2018)	278.690 kDa	(%) Ara: Gal: Man: Rha: Glc = 48.29 ± 2.95: 32.10 ± 1.19: 8.28 ± 0.45: 6.00 ± 0.08: 5.32 ± 0.97	NA	NA	Huang et al. (2018)
	LP (H-2016-a)	370.365 kDa and 8.207 kDa	Rib: Rha: Ara: Xyl: Man: Glc: Gal = 0.45: 0.18: 1.67: 0.3: 1: 1.97: 3.9	NA	NA	Huang et al. (2016a)
	LP (H-2016-b)	NA	Gal: Glc: Man: Ara = 3.90: 1.97: 1.67: 1.00	NA	GC-MS, FT-IR,^13^C NMR, AFM	Huang et al. (2016b)
	LP1	NA	(%) Ara: Glc: Gal = 1.29: 1.00: 1.25	LP1 is composed of (1 → 4,6)-*β*-d-Glc and (1 → 4)-*α*-l-Gal.	GC-MS, FT-IR, NMR	Huang et al. (2016b)
	LP2	NA	(%) Rha: Ara: Gal = 1.00: 2.84: 1.08	LP2 is composed of (1→3)- *α*-l-Ara and (l→2)- *β*-d-Gal.	GC-MS, FT-IR, NMR	Huang et al. (2016b)
	LP3	NA	(%) Rha: Ara: Gal = 1.00: 2.50: 1.09	LP3 is composed of *α*-l Ara and (l→ 4)-*β*-Rha.	GC-MS, FT-IR, NMR	Huang et al. (2016b)
	LCP70S-1	11.7 kDa	Rha: Ara: Gal = 1.06: 6.39: 4.21	The backbone was constructed by (1→3)-linked galactopyranosyl with branches at O-6. The three branches consisted of (1→3)-linked rhamnopyranosyl residues, (1→3,6)-linked galactopyranosyl and (1→5)-linked arabinofuranosyl residues, and each of them was terminated with a (1→)-linked arabinopyranosyl residues.	HPAEC-PAD, GC-MS and^13^C NMR	Hu et al. (2015)
	LP-4	93.214 kDa	(%) Rib: Rha: Ara: Xyl: Man: Glc: Gal = 0.83: 0.29: 1.18: 0.75: 17.25: 75.44: 4.26	NA	NA	Huang et al. (2015)
	LP-6	87.697 kDa	(%) Rib: Rha: Ara: Xyl: Man: Glc:Gal = 1.37: 0.68: 7.16: 2.93: 20.89: 43.23: 23.74	NA	NA	Huang et al. (2015)
	LP-8	87.062 kDa	(%) Rib: Rha: Ara: Xyl: Man: Glc:Gal = 0.86: 1.02: 8.48: 2.89: 23.66: 29.94: 33.15	NA	NA	Huang et al. (2015)
	LPD	370.365 kDa; 8.207 kDa	(%) Rha: Ara: Xyl: Man: Glc: Gal = 1.86: 17.62: 3.21: 10.56: 20.82: 41.18	NA	NA	Huang et al. (2014)
	LPF	970.085 kDa	(%) Rha: Ara: Xyl: Man: Glc: Gal = 0.31: 5.44: 0.71: 15.18: 66.1: 11.58	NA	NA	Huang et al. (2014)
	LCP50S-2	2.19 × 10^2^ kDa	Rha: Ara: Gal: Glc: Xyl = 1.00: 7.30: 3.06: 15.60: 8.18	LCP50S-2 is composed of (1→3)-linked-*β*-l-rhamnopyranosyl residues, (1→4)-linked *α*-d-xylopyranosyl residues, (1→4)-linked *β*-d-glucopyranosyl residues, and (1→4)-linked *α*-d-glucopyranosyl residues, which branched at O-6.	HPAEC-PAD, GC-MS,^13^C NMR	Hu et al. (2011)
	LCP50W	47.2 kDa	Rha: Ara: Gal: Glc: Man = 2.45: 5.78: 1.00: 4.58: 1.32	LCP50W is composed of (1→3)-linked *β*-l-rhamnopyranosyl, (1→6)-linked *α*-d-glucopyranosyl, and (1→2,6)-linked *α*-d-glucopyranosyl residues, which branched at O-6.	GC-MS, NMR spectroscopy analysis	Jing et al. (2014)
	LFP-I	NA	Ara: Rha: Rib: Gal: Glc = 1.95: 2.00: 1.00: 2.04: 1.57	NA	NA	Kong et al. (2010)
	LFP-II	NA	Ara: Rha: Glc = 1.00: 1.20: 1.47	NA	NA	Kong et al. (2010)
	LFP-III	NA	Ara: Rha: Rib: Gal: Glc = 1.30:1.91:1.54:2.13:1.00	NA	NA	Kong et al. (2010)
	LFP-IV	NA	Ara: Rha: Gal: Glc = 1.60: 1.00: 1.07: 1.21	NA	NA	Kong et al. (2010)
	LCPA50-S1	158 kDa	Rha: Ara: Gal: Glc = 1.00: 8.44: 9.96: 1.37	LCPA50-S1 is constructed by (1→3)-*β*-Gal*p*, (1→4)-*β*-Gal*p*, (1→6)-*β*-Gal*p*, (1→6)-*α*-glucans, and (1→4)-*α*-polygalacturonans.	GC-MS, NMR spectroscopy analysis	Wang et al. (2015)
	LPPBa	2,400 kDa	Ara: Rha: Gal: GalA = 2.76: 0.89: 3.02: 1.00	The backbone was constructed by →4)-*α*-d-Gal*p*A6Me-(1→ and →6)-*β*-d-Gal*p*-(1→. The branch chains included *α*-l-Ara*f*-(1→5)-*α*-l-Ara*f*-(1→, *α*-l-Rha*p*-(1→ and *α*-l-Ara*f*-(1→.	HPGPC, NMR spectroscopy analysis, GC	Yang et al. (2016)
The seeds of litchi	LSP-W-4	6.70 kDa	Ara: Man: Glc: Gal = 6.33: 3.88: 10.35: 1.00	Major backbone structure: 1,4-*α*-Glc*p* and 1,4-*β*-Man*p*, Side chain: T-*α*-Gal*p*, T-*α*-Ara*f*, *α*-Ara*f*-(1→5)-*α*-Ara*f*-(1→ and *α*-Ara*f*-(1→5)-*α*-Ara*f*-(1–5)-*α*-Ara*f*-(1→attached to O-6 of 1,4-Man*p* and 1,4-Glc*p*.	HPGPC, FT-IR, GC-MS, NMR spectroscopy analysis	Wu et al. (2020)

Abbreviation: N/A, information was not available.

### 3.1 Monosaccharide composition

**FIGURE 3:**
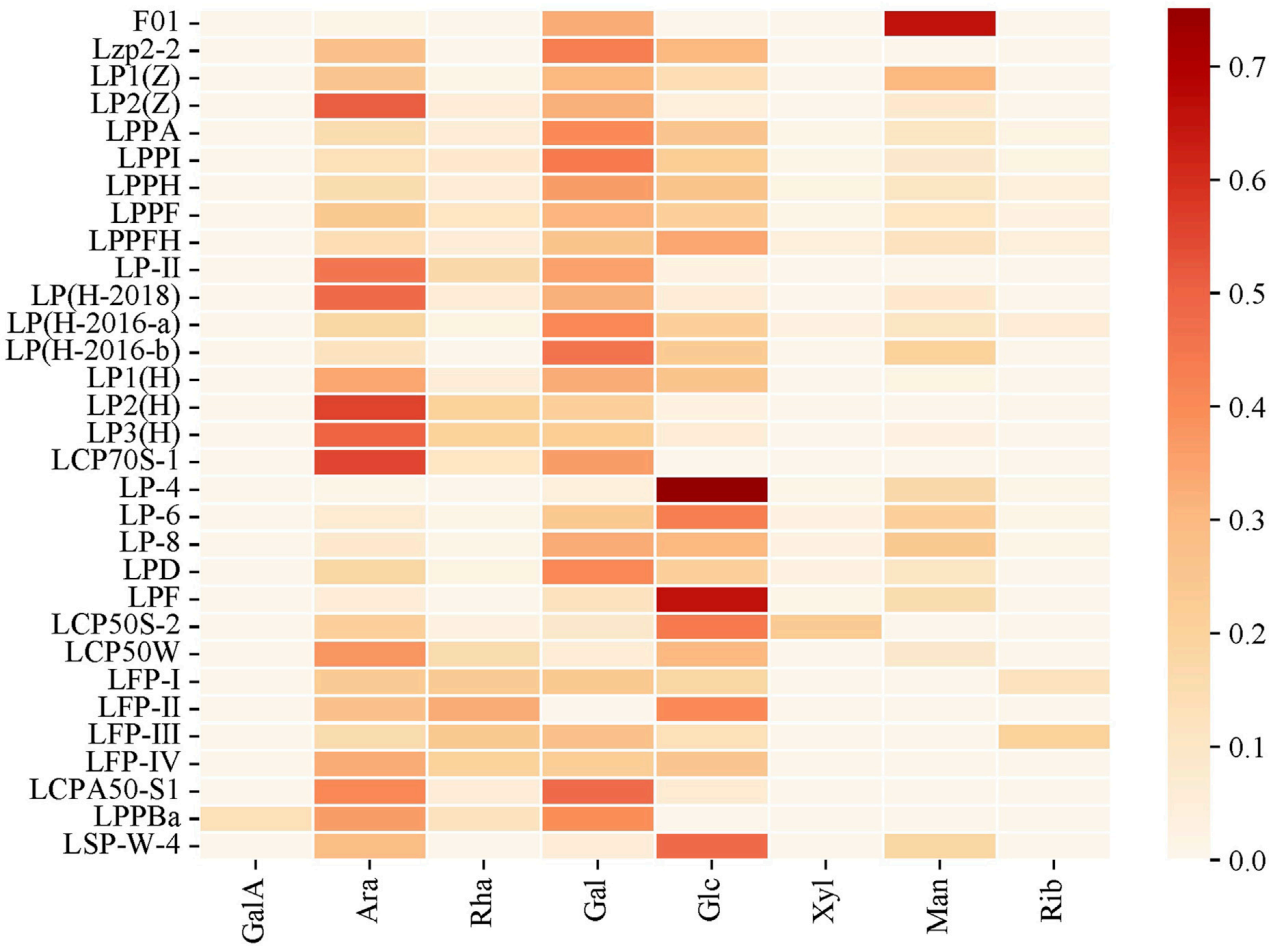
Heat map of monosaccharide composition of litchi polysaccharides.

The monosaccharide composition analysis process of litchi polysaccharides includes hydrolysis, separation, chemical modification, and detection analysis (Chen M. et al., 2024). At present, research on the monosaccharide composition of litchi polysaccharides is mainly focused on the analysis of the types and contents of monosaccharides (Sun et al., 2024). Litchi polysaccharides mainly consist of galactomannan (GalA), arabinose (Ara), rhamnose (Rha), galactose (Gal), glucose (Glc), xylose (Xyl), mannose (Man), and ribose (Rib), existing in varying molar ratios (Figure 3). For instance, acid hydrolysis, acetic acid acetonitrile derivatization, and gas chromatography analysis revealed that LPPBa from litchi pulp is an arabinogalactan with a molar ratio of Ara: Rha: Gal: GalA = 2.76: 0.89: 3.02: 1.00 (Yang et al., 2016). Polysaccharides from litchi pericarp are mainly composed of 65.6% Man, 33.0% Gal, and 1.4% Ara (Yang et al., 2006). The neutral heteropolysaccharide LSP-W-4 isolated and purified from litchi seeds is composed of Ara, Man, Glc, and Gal with a molar ratio of 6.33:3.88:10.35:1.00 (Wu et al., 2020).

### 3.2 Molecular weight

Mw is a key parameter that influences the chemical properties and biological activity of polysaccharides (Sun et al., 2024). Several ways to express the Mw of polysaccharides exist, including Mw, number average molecular weight (Mn), viscosity average molecular weight (Mv), and Z-average molecular weight (Mz) (Wang Z. et al., 2023). The literature generally reports on Mw. The typical methods for determining the Mw of litchi polysaccharides include gel permeation chromatography (GPC) and high-performance GPC (HPGPC). Available data show that the Mw of litchi polysaccharides ranges from 5.54 kDa to 2,400 kDa (Table 2). For example, the Mw of the polysaccharide LSP-W-4 extracted from litchi seeds is 6.70 kDa, while the Mw of LP1 polysaccharide extracted from litchi fruit is 378.67 kDa (Wu et al., 2020; Zou et al., 2023). The considerable variation in the Mw of litchi polysaccharides is likely attributable to differences in the source material, processing methods, and preparation techniques employed in their extraction.

### 3.3 Chemical structure

**FIGURE 4:**
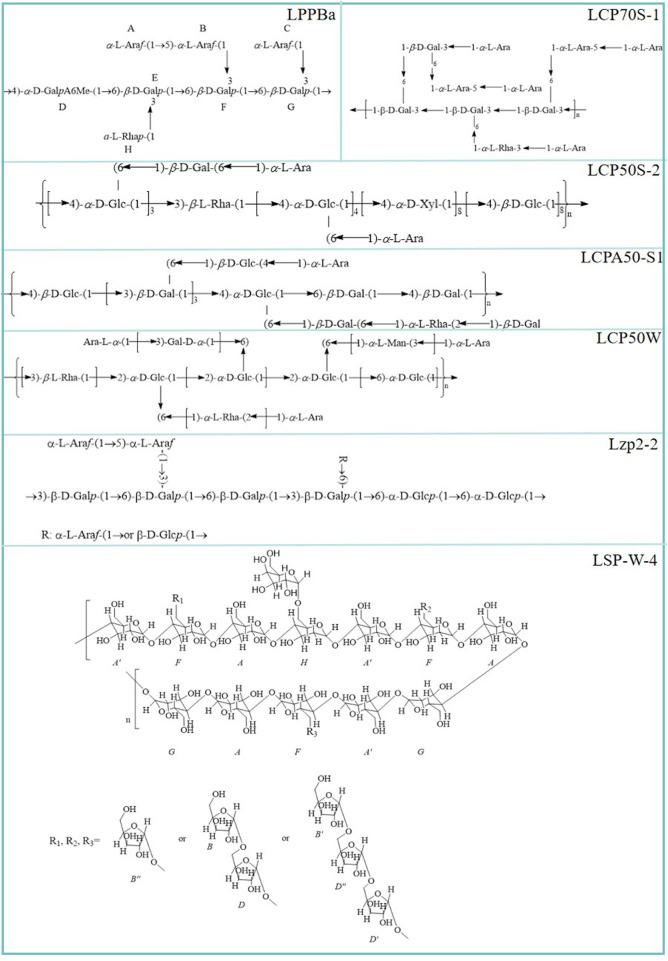
Chemical structure of litchi polysaccharides.

As is well known, studying the chemical structure of polysaccharides is beneficial for elucidating their biological activity and health benefits, and for developing their potential value. Yang et al. (2016) preliminarily identified the precise structure of LPPBa. Its main chain is composed of →4)-*α*-d-Gal*p*A6Me-(1→ and →6)-*β*-d-Gal*p*-(1→. Branch chains include *α*-l-Ara*f*-(1→5)-*α*-l-Ara*f*-(1→*α*-l-Rha*p*-(1→, *α*-l-Ara*f*-(1→). Hu et al. (2011) discovered that the polysaccharide LCP50S-2, extracted from pulp tissues of litchi, consists of a main chain in its structure composed of (1→3)-linked *β*-l-rhamnopyranosyl residues, (1→4)-linked *α*-d-xylopyranosyl residues, (1→4)-linked *β*-d-glucopyranosyl residues, and (1→4) linked *α*-d-glucopyranosyl residues, while the side chain includes *α*-l-arabinopyranosyl residues and (1→6)-linked *β*-d-galactopyranosyl residues. These two side chains are terminated by *α*-l-arabinopyranosyl residues (Hu et al., 2011). Subsequently, the team further studied litchi polysaccharide (LCP50W). The backbone of LCP50W was constructed by (1→3)-linked *β*-l-rhamnopyranosyl, (1→6)-linked *α*-d-glucopyranosyl, and (1→2,6)-linked *α*-d-glucopyranosyl residues, and the branches may contain (1→ 2)-linked *α*-l-rhamnopyranosyl, (1→3)-linked *α*-d-galactopyranosyl, and (1→3)-linked *α*-l-mannopyranosyl residues. Like the side chain terminal structure of LCP50S-2, it terminated by *α*-l-arabinopyranosyl residues (Jing et al., 2014). Wang et al. (2015) extracted a polysaccharide from litchi named LCPA50-S1, whose main chain of its primary structure is composed of (1→4)-linked *β*-d-glucopyranosyl residues, (1→6)-linked *β*-d-galactopyranosyl, (1→3,6)-linked *β*-d-galactopyranosyl residues, (1→4,6)-linked *α*-d-glucopyranosyl residues and branched at O-6, while the side chain includes (1→2)-linked *α*-l-rhamnopyranosyl residues, (1→4)-linked *β*-d-glucopyranosyl residues, and (1→6)-linked *β*-d-galactopyranosyl, terminated with (1→)-linked *α*-l-arabinopyranosyl residues and (1→)-linked *β*-d-galactopyranosyl residues. The proposed structures of LCPA50-S1 are shown in Figure 4.

## 4 Health benefit of litchi polysaccharides

Modern pharmacological research has shown that litchi polysaccharides have hypoglycemic effect, prebiotic effect, promoting effect on exopolysaccharide production by *Weissella confusa*, antioxidant effect, antiproliferative effect, immunoregulatory effect. These health benefits and their potential mechanisms are shown in Table 3.

**TABLE 3:** Summary of *in vitro* biological activities of polysaccharides from litchi.

Health benefit	Polysaccharide name	Testing subjects	Doses/duration	Effects/mechanisms	References
Hypoglycemic effect	LPPA, LPPI, LPPH, LPPF and LPPFH	*α*-glycosidase inhibitory activity assays	0.5–4.0 mg/mL	IC_50_ values (LPPA) = 4.17 mg/mL, IC_50_ values (LPPI) = 3.63 mg/mL, IC_50_ values (LPPH) = 3.28 mg/mL, IC_50_ values (LPPF) = 2.10 mg/mL, IC_50_ values (LPPFH) = 2.53 mg/mL.	An et al. (2022)
	LSP-W-4	*α*-glucosidase inhibition activity assay	0, 0.20, 0.40 and 0.60 mg/mL	IC_50_ (mammalian *α*-glucosidase): 66.97 μM; IC_50_ (yeast *α*-glucosidase): 75.24 μM.	Wu et al. (2020)
Prebiotic effect	LP1 and LP2	Four lactic acid strains: *Lactobacillus plantarum*, *Leuconostoc mesenteroides*, *Lactobacillus casei* and *Bifidobacterium adolescentis*	At a final concentration of 2.0% (w/v)	The number of microorganisms: *Bifidobacterium adolescentis*: FOS > LP2 > LP1. *Lactobacillus casei*: LP2 ≈ FOS > LP1. *Leuconostoc mesenteroides*: LP2>FOS ≈ LP1. *Lactobacillus plantarum*: LP2 > FOS > LP1.	Zou et al. (2023)
	LPs	*B. adolescentis, B. infantis,* and *B. longum*	At a final concentration of 1.0% (w/v)	*B. adolescentis:* At 48 h, LP-6, LP-24, and LP-60 exhibited better prebiotic activity than LP-0. *B. infantis*: LP-6 and LP-24 exhibited better prebiotic activity compared to LP-0. *B. longum*: LP-6 exhibited the most bacterial growth among fermented LPs compared to unfermented LP-0.	He et al. (2022)
Promoting effect on exopolysaccharide production by *Weissella confusa*	Lzp2-2	Yoghurt fermented by *W. confusa*	10 mg/mL	Adding Lzp2-2 can significantly improve the fermentation characteristics and quality of yogurt fermented with *W. confuse.*	Hu et al. (2023)
Antioxidant effect	LCP70S-1 and LCP70W	DPPH radical-scavenging activity, hydroxyl radical-scavenging activity, reducing power assay	DPPH radical-scavenging activity and hydroxyl radical-scavenging activity: 0, 25, 50, 100, 200, 400, 800, and 1,600 μg/mL. Reducing power assay: 0.1–20 mg/mL, 2.5 mL	DPPH radical-scavenging activity: Their maximum value can be up to a percentage of VC: LCP70S-1 = 67.4%, LCP70W = 44.1%. Hydroxyl radical-scavenging activity: Their maximum value can be up to a percentage of VC: LCP70S-1 = 65.0%, LCP70W = 44.6%. Reducing power assay: The reducing abilities of LCP70S-1 (20.0 mg/mL) = 0.63 and LCP70W (20.0 mg/mL) = 0.47.	Hu et al. (2015)
	LCP50S-2	DPPH radical-scavenging activity, hydroxyl radical-scavenging activity	0, 25, 50, 100, 200, 400, and 800 μg/mL	IC_50_ values (DPPH radical-scavenging activity): 220 μg/mL. IC_50_ values (Hydroxyl radical-scavenging activity): 266 μg/mL.	Hu et al. (2011)
	LFP-I, LFP-II, LFP-III and LFP-IV	DPPH radical-scavenging activity, superoxide anion-scavenging activity, hydroxyl radical scavenging activity, ferrous ion chelating ability, reducing power	0–2.0 mg/mL	DPPH radical-scavenging activity: LFP-I (1.0 mg/mL) = 29.2% and LFP-III (1.0 mg/mL) = 34.9%, For LFP-II (1.0 mg/mL) = 25%, LFP-IV (1.0 mg/mL) = 24%. Superoxide anion-scavenging activity: IC_50_ values (LFP-I) = 1.29 mg/mL, IC_50_ values (LFP-III) = 0.87 mg/mL. Hydroxyl radical scavenging activity: IC_50_ values (LFP-I) = 0.81 mg/mL, IC_50_ values (LFP-III) = 0.65 mg/mL. Ferrous ion chelating ability: The chelating ability was 28.57%–91.53%, 20.0%–83.75%, 15.84%–90.50% and 22.45%–81.33% for LFP-I - LFP-IV (0.2–2.0 mg/mL), respectively. Reducing power: The reducing power was 5.50%–29.10%, 10.15%–25.56%, 16.22%–43.89% and 15.12%–23.17% for LFP-I - LFP-IV (0.2–2.0 mg/mL), respectively.	Kong et al. (2010)
	LP-4, LP-6 and LP-8	Oxygen radical absorbance capacity, cellular antioxidant activity assay	100, 200, 400, 800 μg/mL	The ORAC values of the LPFs ranged from 22.08 to 28.14 mol 279 μmol TE/g DW. The order of CCA values from high to low is: LP8 (10.79 mol QE/g DW)>LP4 (9.66 mol QE/g DW)>LP6 (4.72 mol QE/g DW).	Huang et al. (2015)
Antiproliferative effect	LP-4, LP-6 and LP-8	Tumour cell lines A549, HepG2, and MGC-803	100, 200, 400, and 800 μg/mL	The results showed that litchi polysaccharides had inhibitory effects on these three tumour cell lines, with inhibition rates ranging from 10.69% to 38.1%, 9.34%–40.96%, and 4.8%–58.86%, respectively.	Huang et al. (2014)
	LPF and LPD	Tumour cell lines A549, Hela, and HepG2	50–750 μg/mL	The inhibitory effects of LPF and LPD on the proliferation of A549 cells were 2.59%–30.07% and 2.56%–27.17%, respectively. Similarly, at the tested concentration, incubation of Hela cells with LPF and LPD resulted in a growth decrease of 4.61%–28.04% and 5.17%–35.65%, respectively. The inhibition rate of LPF on HepG2 cells ranged from 0.57% to 24.12%. The inhibition rate of LPD on HepG2 cells ranged from 3.11% to 41.37%.	Huang et al. (2014)
Immunoregulatory effect	LCPA50-S1	The spleen of Kunming mice, RAW264.7 cells.	0, 31.3, 62.5, 125, 250, and 500 μg/mL	The concentrations of litchi polysaccharide LCPA50-S1 were 0, 31.3, 62.5, 125, 250, and 500 μg/mL, which significantly promoted spleen lymphocyte proliferation in a concentration dependent manner and also promoted the production of cytokine IL-2. In addition, LCPA50-S1 can rapidly increase NO production and TNF-*α* secretion in RAW264.7 cells after 24 h of stimulation.	Wang et al. (2015)
	LCP50W	The spleen of Kunming mice, YAC-1 lymphoma cell	Splenic lymphocyte proliferation assay: 0–500 μg/mL**.** Natural Killer (NK) cell cytotoxicity assay: 0–250 μg/mL. Measurement of Th1/Th2 cytokines and determination of Th1/Th2 specific transcription factors 0–62.5 μg/mL. Determination of cell cycle distribution:0–125 μg/mL	LCP50W has the potential to enhance mouse immune function, which may be mediated by affecting Th1/Th2 balance.	Jing et al. (2014)
	LP 1–3	MLN cells	25, 50, and 100 μg/mL	At a concentration of 25–100 μg/mL, litchi polysaccharide LP 1–3 can stimulate the proliferation of Mesenteric lymph node (MLN) cells and exhibit dose-dependent secretion of IL-6 and IFN-γ, indicating that they can stimulate MLN cells to regulate the intestinal immune system	Huang et al. (2016b)

### 4.1 Hypoglycemic effect

**FIGURE 5:**
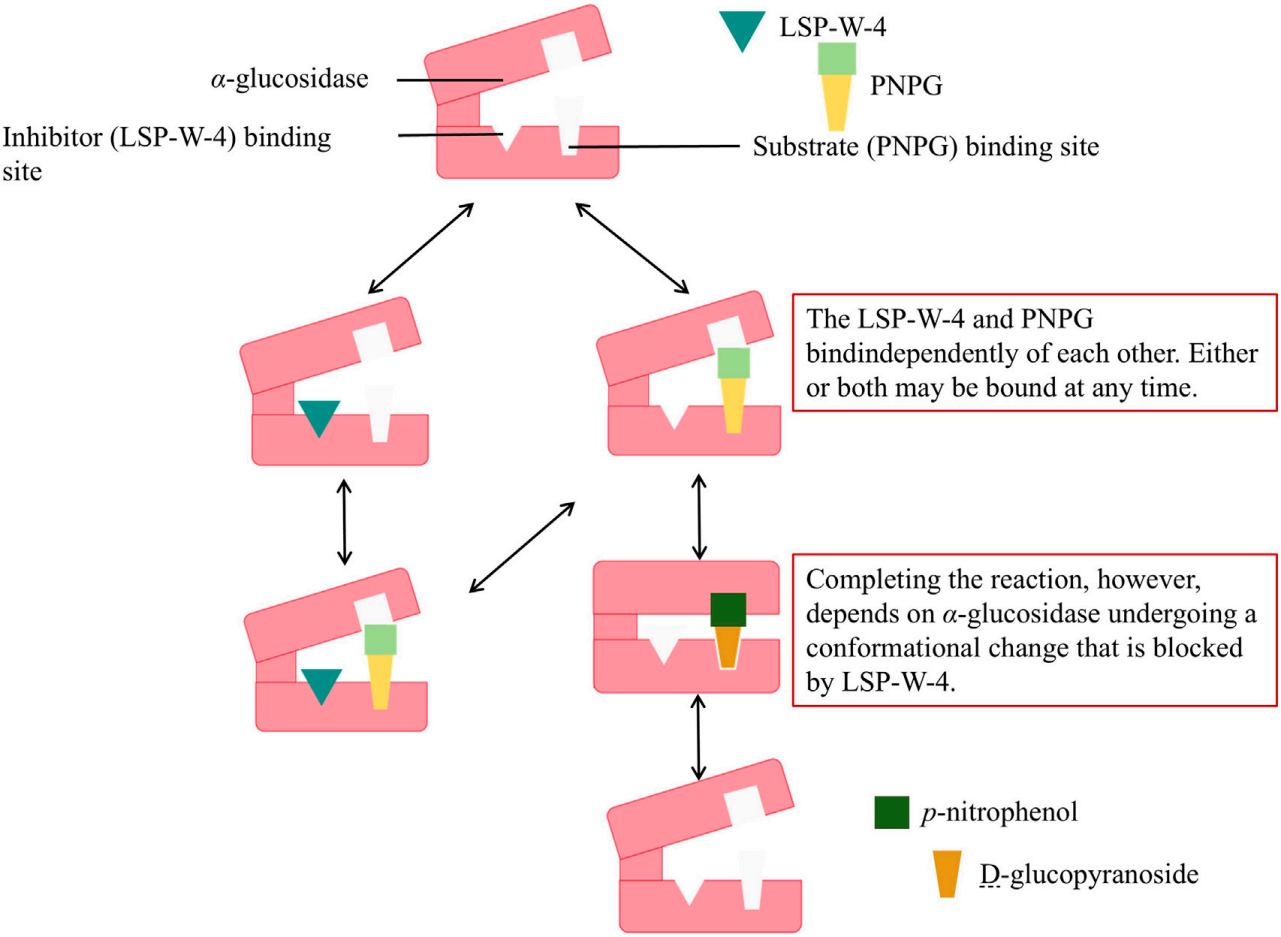
Schematic representation of the molecular mechanism of hypoglycemic effect of litchi polysaccharides.

Diabetes is a chronic disease that severely impairs the body’s health. It has a high incidence rate and can lead to mortality. Diabetes mellitus type 2 (T2DM) is one of the most common types (Khumalo et al., 2024). A hallmark of T2DM is elevated postprandial blood glucose, which is largely influenced by the rate and extent of carbohydrate digestion. *α*-glucosidase is a crucial enzyme for the control of regulating glycoprotein metabolism and plays a vital role in carbohydrate digestion and absorption (Benjamin et al., 2024). It has become a promising therapeutic target for relieving postprandial hyperglycemia. Research has indicated that litchi polysaccharides inhibit *α*-glucosidase activity, thereby delaying carbohydrate absorption and lowering postprandial blood glucose levels (El-Nashar et al., 2024). To further explore the effects of litchi polysaccharides on *α*-glucosidase, Wu et al. (2020) investigated the inhibitory activity of litchi polysaccharide (LSP-W-4) against *α*-glucosidase from two sources: rat intestines and yeast (*Saccharomyces cerevisiae*). The study also examined the interaction between LSP-W-4 and *α*-glucosidase through kinetic enzymatic analysis. The results showed that LSP-W-4 effectively inhibited both yeast- and mammalian-derived *α*-glucosidases, with the most notable difference in IC_50_ between yeast-derived *α*-glucosidases (66.97 μM) and mammalian-derived ones (75.24 μM). The addition of varying concentrations (0, 0.20, 0.40, and 0.60 mg/mL) of LSP-W-4 led to a linear change in absorbance corresponding to *α*-glucosidase concentration (0.025, 0.050, 0.100, and 0.200 U/mL). Notably, the slope of the line decreased with lower LSP-W-4 concentrations, indicating that LSP-W-4 interacts with *α*-glucosidase through non-covalent interactions, allowing easy removal *via* dilution or dialysis. These kinetic enzymatic analysis results suggest that LSP-W-4 inhibits *α*-glucosidase activity in a non-competitive manner, with K_M_ values of 0.43 mmol/L and 0.53 mmol/L for yeast and mammalian enzymes, respectively (Figure 5). These findings could provide valuable insights for the development of functional foods targeting diabetes and its complications. In addition to its effects on *α*-glucosidase, litchi polysaccharides also inhibit *α*-amylase. Experimental results revealed that litchi polysaccharides (LPPA, LPPI, LPPH, LPPF, and LPPFH) obtained through five different drying methods exhibited a dose-dependent inhibitory effect on *α*-amylase within the concentration range of 0–4.0 mg/mL. The inhibitory activity followed the order: LPPF > LPPFH > LPPH > LPPI > LPPA. Overall, the hypoglycemic activity of litchi polysaccharides, as evidenced by existing research, is primarily attributed to their inhibition of *α*-glucosidase (An et al., 2022).

### 4.2 Prebiotic effect

Plant polysaccharides (excluding starch) are resistant to digestion in digestive fluids such as saliva, gastric juice, and intestinal fluid. Polysaccharides that cannot be hydrolyzed by the host’s enzymes pass into the intestine, where they serve as a primary carbon source for the gut microbiota. These polysaccharides can exert health benefits by selectively promoting the proliferation of beneficial gut probiotics (Zhang et al., 2022; Bock et al., 2024). Litchi polysaccharides, as a type of plant polysaccharide, possess prebiotic activity. Experimental studies on prebiotic effects have revealed that litchi polysaccharides promote the growth of various probiotic strains, including *Lactobacillus plantarum*, *Leuconostoc mesenteroides*, *Lactobacillus casei*, *Bifidobacterium adolescentis*, *Bifidobacterium infantis*, and *Bifidobacterium longum*. Zou et al. (2023) investigated the prebiotic activity of litchi polysaccharides LP1 and LP2 using four lactic acid bacteria strains: *L. plantarum*, *Leuconostoc enterica*, *L. casei*, and *B. adolescentis*. The results demonstrated that the addition of litchi polysaccharides promoted the growth of all four bacterial strains. Notably, different carbon source media significantly influenced the growth of *L. mesenteroides* (*P* < 0.05). LP2 exhibited strong prebiotic activity, enhancing the growth of *L. mesenteroides*, followed by fructooligosaccharides (FOS) and LP1. For *L. plantarum*, the growth-promoting effects of the three carbon sources were similar, with LP2 > FOS > LP1. The proliferation of *B. adolescentis* was also highest with LP2, followed by FOS and LP1. While the microbial growth of *L. casei* was comparable between FOS and LP2, LP1 showed the least effect (*P* < 0.05). These findings suggest that LP2 is more effective than LP1 in promoting probiotic growth, but further research is needed to elucidate the underlying mechanisms. He et al. (2022) investigated the prebiotic activity of litchi polysaccharides using three strains of *Bifidobacterium* through an *in vitro* fermentation experiment over different time intervals (0–72 h). The study used carbohydrate-free MRS broth with 0.05% (w/v) L-cysteine as the base medium, with FOS serving as the positive control. The results showed that fermentation time influenced the effect of litchi polysaccharides on the proliferation of the *Bifidobacterium strains*. Among the polysaccharides tested, LP-6 exhibited the most significant prebiotic effect by promoting the growth of *Bifidobacterium* strains (He et al., 2022).

### 4.3 Promoting effect on exopolysaccharide production by *Weissella confusa*

Microbial polysaccharides can be classified into interstitial polysaccharides and exopolysaccharide based on their location in cells. Exopolysaccharides (EPS) have adhesive properties, thickening properties, and water and oil retention. They can be used as stabilizers, emulsifiers, thickeners, and applied in food processing production (Salimi and Farrokh, 2023). Research shows that litchi polysaccharides can promote the production of EPS by *Weissella confusa* (*W. confusa*). An experiment evaluated the effect of Lzp2-2 on fermented yogurt through its physicochemical properties and flavor. Adding Lzp2-2 (10 mg/mL) to the yogurt fermentation medium, the viscosity of the yogurt gradually increased with the extension of fermentation time (0, 8, 24, 48, 72 h). At equivalent amount of sucrose, the viscosity of the sucrose treatment group was lower than that of the Lzp2-1 treatment group. The EPS content in the Lzp2-2 treatment group was higher than that in the sucrose treatment group. This may be because Lzp2-2 promotes the production of EPS by *W. confusa*. EPS and milk protein interact to form gel network, resulting in increased viscosity of yogurt. In addition, Lzp2-2 can be hydrolyzed by glycoside hydrolases into various monosaccharides such as Gal, Glc, and Ara. Subsequently, Gal, Glc, and Ara simultaneously participate in the biosynthesis of EPS through the Wzx/Wzy dependent pathway, ABC transporter pathway, synthase dependent pathway, and sucrose-synthase dependent pathway. In the control group, only Glc in sucrose participated in the synthesis of EPS by *W. confusa*. So, there are differences in viscosity at the same time point. The structural characteristics of yogurt, including hardness, adhesion, adhesiveness, chewiness, and elasticity, are considered important indicators of its overall acceptability. After adding Lzp2-2, these indicators significantly improved, while the difference in cohesion was not significant. In addition, the effect of Lzp2-2 on yogurt flavor was detected using electronic nose and electronic tongue. The Lzp2-2 treatment group increased the content of sulphur, ketones, alcohols, aldehydes, nitroxide, and short-chain alkanes in yogurt. And the richness, sweetness, freshness, and acidity of the Lzp2-2 treatment group were higher than those of the control group. These may all be related to the generation of EPS by *W. confusa*. In summary, adding litchi polysaccharides can significantly improve the fermentation characteristics and quality of yogurt fermented with *W. confusa*, providing scientific reference for the rational application of litchi polysaccharides in the production of new dairy products and theoretical basis for the high value-added utilization of litchi resources (Hu et al., 2023).

### 4.4 Antioxidant effect

Free radicals are clusters of atoms that contain one unpaired electron. Studies have shown that the occurrence, development, and prognosis of various related diseases such as heart, liver, kidney, and brain are closely related to the excessive production of free radicals or the decrease in the ability to scavenge free radicals. Antioxidants play a pivotal role in capturing and neutralizing free radicals, making them essential in defending against free radical-related diseases (Huang et al., 2017; Zhou et al., 2022). Litchi polysaccharides exhibit significant antioxidant activity, characterized by low cost and minimal side effects, positioning them as a promising focus for antioxidant research.

The antioxidant capacity of litchi polysaccharides has been evaluated using several common assays, including DPPH, ABTS, hydroxyl radical scavenging, superoxide anion radical scavenging, and reducing ability tests. Studies have shown that litchi polysaccharides, such as LCP50S-2, LCP70S-1, and LCP70W, demonstrate increased scavenging effects on DPPH and hydroxyl radicals with rising concentrations. In the DPPH radical scavenging assay, the maximum scavenging percentages of these polysaccharides reached 75.6%, 67.4%, and 44.1% of the positive control (Vitamin C), respectively. In the hydroxyl radical scavenging assay, the values were 73.9%, 65.0%, and 44.6%, respectively (Hu et al., 2011; Hu et al., 2015). Four polysaccharides (LFP-I, LFP-II, LFP-III, and LFP-IV) isolated and purified from litchi pulp tissue showed varying levels of DPPH radical scavenging at a concentration of 1.0 mg/mL. The scavenging ability followed the order: LFP-III > LFP-I > LFP-II > LFP-IV. At concentrations ranging from 0.1 to 2.0 mg/mL, the superoxide anion scavenging effects were 18.62%–67.24% for LFP-III, 17.10%–62.07% for LFP-I, 10.28%–46.24% for LFP-II, and 9.62%–44.24% for LFP-IV. In the hydroxyl radical scavenging assay, the IC_50_ values for LFP-I and LFP-III were found to be 0.81 mg/mL and 0.65 mg/mL, respectively. In the Ferrous ion chelating assay, the chelation ability of each LFP increases with the increase of concentration. There is a good correlation between the reducing ability of all LFPs and the increase in concentration. After comprehensive consideration and analysis of existing results, LFP-III has the strongest antioxidant capacity and is worthy of further research (Kong et al., 2010). In addition, due to the closer proximity of cells to the internal environment of organisms, using cell models for antioxidant activity (CAA) research is also a commonly used method. Huang et al. (2015) compared the antioxidant activity of three litchi polysaccharides (LP4, LP6, and LP8) using an 2,2′azobis-(2-amidinopropane)-hydrochloride (AAPH) induced oxidative stress model in HepG2 cells. The order of CCA values from high to low is: LP8 (10.79 mol QE/g DW) > LP4 (9.66 mol QE/g DW) > LP6 (4.72 mol QE/g DW). The content of galactose and mannose in LP8 is higher than that in LP4 and LP6, which is the reason why it has strong cellular antioxidant activity.

### 4.5 Antiproliferative effect

Cancer is one of the leading causes of death. It not only has a direct harm to the patient’s body but also has a profound impact on the patient’s psychological and social life. Therefore, cancer prevention, early detection and treatment are particularly important. At present, the main methods for treating cancer are chemotherapy and radiotherapy (Cao et al., 2014). But these methods are susceptible to the effects of tumor cell drug resistance or radiation resistance and may have varying degrees of side effects on patients during the treatment process. Plant polysaccharides, as natural biomacromolecules, are considered promising candidate drugs for treating cancer due to their excellent anti-cancer activity and small side effects and have received increasing attention in recent years (Wang A. et al., 2023). Huang et al. (2014) investigated the inhibitory effect of litchi polysaccharides on the proliferation of tumor cells. Using tumor cell lines A549, HepG2, and MGC-803 as cell models, the *in vitro* anti-proliferative activity of litchi polysaccharides (100, 200, 400, and 800 μg/mL) on these three types of cells was examined. The results showed that litchi polysaccharides exhibited significant inhibitory activities against the three tumor cell lines, with inhibition rates varying between 10.69% and 38.1%, 9.34% and 40.96%, and 4.8% and 58.86%, respectively (Huang et al., 2015). The inhibitory effect of litchi polysaccharides (50–750 μg/mL LPF and LPD) on tumor cell lines A549, Hela, and HepG2 have been studied. The inhibitory effect of LPF and LPD on proliferation of A549 cells was 2.59%–30.07% and 2.56%–27.17%, respectively. Similarly, cell growth was inhibited by 4.61%–28.04% and 5.17%–35.65% when Hela cells were incubated at the tested concentrations with LPF and LPD, respectively. The inhibition rate of LPF on HepG2 cells was 0.57%–24.12%. The inhibitory percentage of LPD against HepG2 cells ranged from 3.11% to 41.37%. Briefly, both above two litchi polysaccharides have the function of inhibiting the growth of A549, HeLa, and HepG2 cells in a concentration-dependent manner (Huang et al., 2014). The experiments below suggest that litchi polysaccharides have potential for use in chemoprevention and tumor therapy; however, the specific mechanism of action requires further study.

### 4.6 Immunoregulatory effect

**FIGURE 6:**
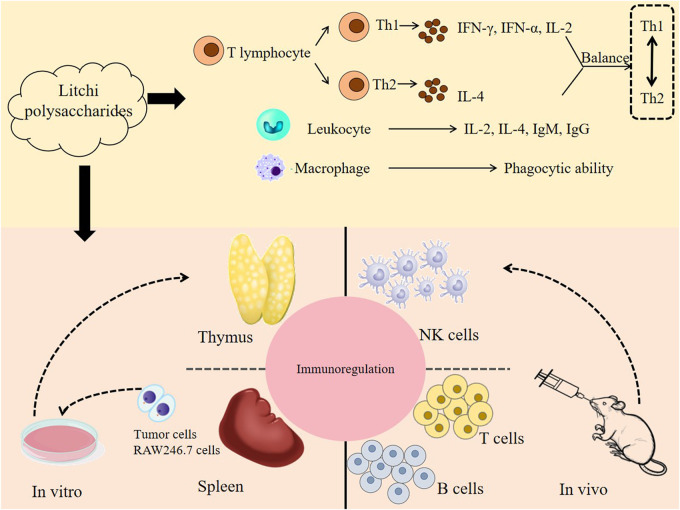
Schematic representation of the molecular mechanism of immunoregulatory effect of litchi polysaccharides.

Immunity is generally considered the primary protective mechanism against various diseases (Mirzadeh, M., Keshavarz Lelekami, A., & Khedmat, L., 2021). What current research indicates is that litchi polysaccharides play an important role in enhancing the body’s immune system, predominantly by affecting what might be characterized as immune cells and cytokines (Figure 6). Splenic lymphocyte proliferation tends to represent an important indicator to study immune response and evaluate cell activity. Wang et al. (2015) found that the concentrations of litchi polysaccharide LCPA50-S1 were 0, 31.3, 62.5, 125, 250, and 500 μg/mL, which significantly promoted spleen lymphocyte proliferation in a concentration dependent manner and promoted the production of cytokine IL-2. In addition, considering the nuanced nature of these findings, LCPA50-S1 rapidly increased NO production and TNF-*α* secretion in RAW264.7 cells after 24 h of stimulation. These results suggest that LCPA50-S1 may function as an immune modulator for the treatment of various immune-related diseases (Wang et al., 2015). The evidence reveals that litchi polysaccharide LCP50W can typically promote splenic lymphocyte proliferation at concentrations of 3.9–125 μg/mL. At a concentration of 3.9–250 μg/mL, NK cell killing activity can also seemingly be enhanced in what appears to be a concentration-dependent manner. IFN-γ and IL-4 are important cytokines that regulate the body’s immune response. LCP50W promotes the secretion of Th1 cytokine IFN-γ while inhibiting the secretion of Th2 cytokine IL-4. Further exploration of its mechanism reveals that LCP50W enhances the expression of T-bet and predominantly inhibits the expression of GATA-3. What can be reasonably inferred from these findings is that LCP50W possesses the potential to improve mouse immune function, which may be mediated by an effect on the Th1/Th2 balance (Jing et al., 2014). The intestine not only plays a crucial role in the process of digestion and absorption but can also be described as the largest immune organ in the body. Intestinal immunity provides a crucial pathway through which litchi polysaccharides exert their immunomodulatory effects. At a concentration of 25–100 μg/mL, litchi polysaccharide LP 1-3 can stimulate the proliferation of Mesenteric lymph node (MLN) cells and exhibit dose-dependent secretion of IL-6 and IFN-*γ*, indicating that they can stimulate MLN cells to regulate the intestinal immune system (Huang et al., 2016b).

## 5 Structure-activity relationship of litchi polysaccharides

The biological activity of polysaccharides is closely linked to their chemical structure (Chen N. et al., 2024). Zou et al. (2023) suggests through their investigation of the prebiotic activity of litchi polysaccharides is that what appears to represent the chemical structure of polysaccharides tends to influence their utilization by probiotics. The monosaccharide composition affects the prebiotic activity of litchi polysaccharides (Wang G. et al., 2024; Zhang C. et al., 2024). Compared to LP1, LP2 contains a higher proportion of Ara and Gal contributing to its stronger prebiotic effect. Additionally, the Mw of polysaccharides impacts their accessibility to probiotics. LP1 has an Mw of 378.67 kDa, while LP2 has a lower Mw of 16.96 kDa (Zou et al., 2023). Polysaccharides with smaller Mw tend to have higher solubility and lower viscosity, making them more readily available for probiotic utilization. Polysaccharides from litchi pulp, such as LP1-3, exhibit significant differences in their chemical structure and immunoregulatory activity. This effect can be attributed to their chemical composition, including heteropolysaccharides like (1→4,6)-*β*-d-Glc and (1→4)-*α*-l-Gal, their Mw, and their hyper-branched spherical chain conformation, which enhances recognition by immune cell surface receptors (Huang et al., 2016b). The relationship between the structure of polysaccharides and their pharmacological effects is highly complex, reflecting the influence of multiple structural features on the pharmacological activities of litchi polysaccharides.

Through appropriate structural modifications, the spatial structure, types, numbers of substituents, Mw, and other properties of polysaccharides can be altered, potentially enhancing their biological activity and even generating new biological functions. Common methods for modifying polysaccharides include carboxymethylation, sulfation, phosphorylation, and selenization, all of which significantly influence their antioxidant properties and other biological activities. These modifications expand the practical applications of polysaccharides in areas such as medicine, functional foods, and cosmetics (Liu T. et al., 2023; Zhao et al., 2023). Fermentation is another important method for modifying polysaccharides (Wang A. et al., 2023). Research has shown that fermentation is an effective means of enhancing the prebiotic activity of polysaccharides. He et al. (2022) investigated the chemical structure and prebiotic potential of LPs fermented over different durations. The yield of LPs and the content of uronic acid showed an irregular trend with fermentation time, with a decrease in total sugar content and an increase in Mw. Among the various fermentation times, LP-6 exhibited the highest yield (2.67%) and the most pronounced prebiotic effect, particularly in promoting *Bifidobacterium* growth. This could be attributed to LP-6 having the lowest Mw among all the LPs, and its composition, mainly consisting of Glc, Gal, and Xyl, with the highest content of these sugars, which interact to influence its probiotic activity. The study suggests that the optimal fermentation time is crucial for improving probiotic effects (He et al., 2022). However, there is still significant controversy regarding the mechanism of polysaccharide structural modification, due to the complex structure of polysaccharides. The research on structural modification of polysaccharides still has a long way to go, and it also has special and important significance in the study of polysaccharides.

## 6 Conclusion and prospect of litchi polysaccharides

This article summarizes the existing research results on litchi polysaccharides. Litchi polysaccharides are mainly obtained from the flesh and seeds of litchi. Significant advancements have been made in the extraction and purification processes, yet optimizing extraction conditions to enhance polysaccharide yield remains an area of active investigation. In terms of structural characteristics, research has mostly focused on the determination of primary structural features such as molecular weight and monosaccharide composition, and there are certain differences in the results obtained by different extraction and purification methods. Pharmacologically, litchi polysaccharides have demonstrated a wide range of bioactivities, with particular emphasis on their hypoglycemic effects.

A comprehensive review of litchi polysaccharides has unveiled exciting opportunities for further research and application. However, several pressing challenges must be addressed to facilitate their deeper utilization and commercial application. During the extraction process, litchi polysaccharides may interact with polyphenols or proteins, resulting in complex compounds referred to as “litchi polysaccharide conjugates.” The interactions between polysaccharides and polyphenols, as well as polysaccharides and proteins, likely enhance the biological activity of litchi polysaccharides, particularly in their ability to regulate gut microbiota and exhibit prebiotic functions (Xue et al., 2024). Additionally, these conjugates play a significant role in influencing the taste and stability of products (Liu et al., 2024). However, the formation of these conjugates complicates our understanding of the physicochemical properties of litchi polysaccharides. This highlights the critical need for developing an environmentally friendly and efficient method for preparing litchi polysaccharides. Such methods would not only improve yield but also provide high-quality samples for studying the chemical structure of these polysaccharides. Research into the chemical structure of litchi polysaccharides has spanned nearly two decades, with significant advances in technology over time. Early chromatographic and spectroscopic techniques, with limited resolution and sensitivity, made it challenging to accurately analyze the fine structure of litchi polysaccharides. This makes it difficult to unify the chemical structure of litchi polysaccharides at a certain level. Given the complexity of the theoretical relationships involved, the inconsistency in the depth of research presents a challenge to fully understanding the connection between the structure and activity of litchi polysaccharides. These findings underscore the need for establishing a standardized procedure for the extraction, purification, and characterization of litchi polysaccharides, which could streamline development efforts and enhance research efficiency. Notably, most existing studies on the activity of litchi polysaccharides primarily focus on *in vitro* experiments. Animal studies are essential for validating the pharmacological effects of litchi polysaccharides. Such experiments are necessary to comprehensively assess the potential of these polysaccharides. The dosage range and administration regimen determined through animal experiments can be adjusted more accurately in human clinical trials. Subsequently, on the basis of animal experiments, further human experiments can be conducted to comprehensively evaluate the actual effectiveness and safety of litchi polysaccharides in humans. On this basis, developing litchi polysaccharides as functional foods will be safer and more effective.

In light of these challenges, future research is expected to delve deeper into the complex structural characteristics of litchi polysaccharides. By improving these polysaccharides through chemical synthesis and modification, and further exploring the relationship between their structure and biological activity, researchers will gain important insights that could unlock the full developmental potential of litchi polysaccharides.

## Abbreviations

Ara, Arabinose; CE, Capillary electrophoresis; CAA, Cell models for antioxidant activity; T2DM, Diabetes mellitus type 2; EPS, Exopolysaccharide; FOS, Fructooligosaccharide; Gal, Galactose; GC, Gas chromatography; GPC, Gel permeation chromatography; Glc, Glucose; HPAEC, High-performance anion-exchange chromatography; HPGPC, High-performance gel permeation chromatography; HPLC, High performance liquid chromatography (HPLC); HWE, Hot water extraction; Man, Mannose (Man); MS, Mass spectrometry; MLN, Mesenteric lymph node; Mn, Number average molecular weight; PAD, Pulsed amperometric detection; RI, Refractive index; Rha, Rhamnose; Rib, Ribose; SBE, Semi-bionic extraction; UV, Ultraviolet (UV); Mv, Viscosity average molecular weight; Mw, Weight average molecular weight; XRD, X-ray diffraction; Xyl, Xylose; Mz, Z-average molecular weight.
